# Epithelial-mesenchymal transition couples with cell cycle arrest at various stages

**DOI:** 10.1101/2025.02.24.639880

**Published:** 2025-02-28

**Authors:** Sophia Hu, Yong Lu, Gaohan Yu, Zhiqian Zheng, Weikang Wang, Ke Ni, Amitava Giri, Jingyu Zhang, Yan Zhang, Kazuhide Watanabe, Guang Yao, Jianhua Xing

**Affiliations:** 1 Department of Computational and Systems Biology, University of Pittsburgh, USA.; 2 Joint CMU-Pitt Ph.D. Program in Computational Biology, University of Pittsburgh, USA.; 3 Department of Physics and Astronomy, University of Pittsburgh, USA; 4 CAS Key Laboratory for Theoretical Physics, Institute of Theoretical Physics, Chinese Academy of Sciences, Beijing 100190, China.; 5 School of Physical Sciences, University of Chinese Academy of Sciences, Beijing 100049, China.; 6 RIKEN Center for Integrative Medical Sciences Yokohama, Japan.; 7 Department of Molecular & Cellular Biology, University of Arizona, Tucson, AZ 85721, USA.; 8 Arizona Cancer Center, University of Arizona, Tucson, AZ 85719, USA.; 9 UPMC-Hillman Cancer Center, University of Pittsburgh, USA

**Keywords:** EMT, cell cycle arrest, coupling, dynamical systems theory, single-cell RNA-seq

## Abstract

Numerous computational approaches have been developed to infer cell state transition trajectories from snapshot single-cell data. Most approaches first require projecting high-dimensional data onto a low-dimensional representation, raising the question of whether the dynamics of the system become distorted. Using epithelial-to-mesenchymal transition (EMT) as a test system, we show that both biology-guided low-dimensional representations and stochastic trajectory simulations in high-dimensional state space, not representations obtained with *brute force* dimension-reduction methods, reveal multiple distinct paths of TGF-β-induced EMT. The paths arise from coupling between EMT and cell cycle arrest at either the G1/S, G2/M or M checkpoints, contributing to cell-cycle related EMT heterogeneity. The present study emphasizes that caution should be taken when inferring transition dynamics from snapshot single-cell data in two- or three-dimensional representations, and that incorporating dynamical information can improve prediction accuracy.

## Introduction

Throughout the lifetime of every cell, there are critical moments when they encounter intrinsic and extrinsic signals that direct them towards a specific cellular fate. These decisions occur in various contexts, such as embryonic development, wound healing, cellular senescence, and aging. Within a cell multiple cellular processes function simultaneously; therefore, the decision and execution of a cell fate change often requires coordination between different cellular programs to allocate limited resources and to minimize interference or conflict.

A central, yet long-standing question in cell biology revolves around how the cell cycle couples to and influences other cell fate decisions such as apoptosis, differentiation, and reprogramming. The cell cycle is a tightly regulated cellular process, where a proliferative cell cycles through a temporally ordered G1→S→G2→M sequence, with multiple checkpoints deciding on the continuation or halting of the cell cycle to ensure proper cell division.

The cell cycle couples with cell fate change in a context dependent manner. In the case of terminal differentiation, evidence has shown that cells typically withdraw from the cell cycle and enter an arrested state in order to proceed toward a specific cell fate^1^. In other cases, cell division is required to allow for cell fate switching where the period between the end of mitosis and entry into G1 is a prime window for transcriptional and epigenomic changes to lead to a change in cell fate. The Rb-E2F network, for example, integrates signals from regulators of other cellular processes to decide whether a newly divided cell proceeds through to the G1 phase or exits the cell cycle and enters a quiescent G0 phase^2,3^. In yet other cases, the specific cell cycle phase, G1, S, or G2, a cell resides in can dictate a cell’s fate.

The coupling between the cell cycle and cell fate changes has been exploited to modulate cell fate transitions, such as accelerating the reprogramming process and improving the efficacy of cancer treatment^4,5^. However, the exact mechanisms that govern cell fate decisions, specifically the timing along the cell cycle, remain elusive.

Addressing this question is a formidable challenge as it requires unraveling the regulation mechanisms and transition dynamics of cell phenotypic transition processes which involve many molecular species that are coupled together. The development of single-cell RNA sequencing (scRNA-seq) technology has revolutionized the field of biology by providing genome-wide snapshots of gene expression profiles in heterogeneous cell populations^6,7^. Numerous computational approaches have been developed to infer phenotype transition trajectories from snapshot single-cell data. Pseudotime analysis is one class of computational approaches, where sampled single-cell data are ordered based on their progression through a biological process determined by gene expression similarities^8^. Methods such as optimal transport infer cell state transition dynamics from temporal distributions in the cell state space^9^.

Another class of computational methods integrates single-cell expression states with dynamical information for trajectory inference. In a seminal study, La Manno et al. took advantage of the ability to distinguish unspliced and spliced mRNA transcripts from scRNA-seq data, and estimated instantaneous RNA velocity, the speed and direction of change of a high-dimensional vector in the cell expression space^10^. By taking these instantaneous RNA velocities, estimated using the original splicing-based method or by other improved methods^11^ as input, Qiu et al. developed *dynamo*, a machine learning framework that reconstructs a set of multi-dimensional, analytical, and generally nonlinear vector field function encoding the genome-wide gene regulation relations of the system^12^. Zhang et al. further developed *graph-dynamo* for complete dynamical model studies^13^.

In general, many of the existing dynamics-inference methods are performed on a low-dimensional (e.g., two- or three-dimensional) manifold of the single-cell data^8^. That is, one implicitly assumes that the low-dimensional representation faithfully distinguishes different cell states in a biologically meaningful manner. However, this assumption needs to be carefully evaluated, as inappropriate representations may lead to a distortion of the underlying cellular dynamics, which we will later discuss, and possibly imprecise conclusions.

While preliminary analysis in low dimensional space can aid in visually understanding scRNA-seq data, more emphasis needs to be placed on performing higher dimensional analysis to corroborate conclusions made from low dimensional analysis. In the case of *dynamo*, similar to molecular dynamics simulations which use a force field to describe molecular interactions, with *dynamo* one can perform deterministic or stochastic simulations with the learned vector fields to generate single-cell trajectories in a high-dimensional (dimensions > 20) cell state space to better understand the dynamics within the data.

In this study, we will use a combination of low and high-dimensional methods to study the dynamics of epithelial-to-mesenchymal transition (EMT) using snapshot single-cell RNA-seq data. EMT is an example of a cell phenotype transition that has attracted extensive attention in recent years, due to its involvement in many biological processes including development, wound healing, tissue fibrosis, and cancer metastasis. During EMT, epithelial cells, characterized by an apical-basal polarity and adherence to a basement membrane and other epithelial cells, lose their epithelial traits and develop mesenchymal traits, characterized by a detachment from the basement membrane, an elongated shape and increased mobility^14,15^. The onset of EMT is regulated by a core regulatory network involving transcription factors and microRNAs. These regulators are also involved in other cellular processes like proliferation and regulation of the cell cycle^16-18^.

The coupling between the cell cycle and EMT is well documented in the literature. In the context of human oral squamous cell carcinoma cells treated with TGF-β to induce EMT, TGF-β has been found to induce G1 phase cell cycle arrest^19^. In the case of kidney fibrosis and pancreatic ductal adenocarcinoma, a link between cell cycle arrest and partial EMT has been established where *Snai1*-induced partial EMT also induces G2/M phase cell cycle arrest^20,21^. The discrepancy in the reports raises the question of whether the coupling between the cell cycle and EMT is context dependent.

An open question surrounding EMT is whether EMT proceeds through a single or multiple paths^15^. Pseudo-time analyses of scRNA-seq data of human mammary epithelial MCF10A cells treated with TGF-β, to induce EMT, concluded that EMT proceeds through a one-dimensional continuum^22^. Markov transition model analyses on snapshot proteomic data of non-small-cell lung carcinoma cell lines treated with TGF-β, to induce EMT, also reached a similar conclusion^23^. On the other hand, Wang et al. performed live-cell imaging studies of TGF-β induced EMT in a human lung carcinoma epithelial cell line, A549, with endogenous vimentin-RFP labeling in a multi-dimensional composite cell feature space^24,25^ and found at least two parallel paths that connect the initial epithelial state to the final mesenchymal state.

Live-cell imaging provides direct evidence of cells undergoing a transition process, while pseudo-time trajectory inference methods rely on snapshot scRNA-seq data to group cells with similar gene expression and infer their progression. The discrepancy between the number of identified paths between pseudo-time trajectory inference methods and live-cell imaging methods emphasizes the need for computational methods that can reveal the molecular nature of EMT on a granular single-cell level.

Here, to understand EMT dynamics, we performed vector field analyses using *dynamo* on scRNA-seq data of MCF10A cells treated with increasing concentrations of TGF-β to induce EMT. To better observe the coupling between the cell cycle and EMT, we constructed a pipeline to obtain a cell cycle informed representation. With this representation we predicted multiple cell cycle-EMT paths, corresponding to either the G1/S, G2/M or M cell cycle checkpoint. Using trajectory simulations and transition path analyses we confirmed these paths in high-dimensional space and identified the paths in two other time course scRNA-seq datasets of cells undergoing TGF-β induced EMT. Overall, this study identifies multiple EMT paths involving the coupling between the cell cycle and EMT, advancing our knowledge in the paths cells undergoing EMT can take, and highlighting that cautions should be taken when analyzing scRNA-seq in low-dimensional space.

## Results

### scRNA-seq data of TGF-β induced EMT in MCF10A cells reveals an EMT continuum.

We first analyzed a scRNA-seq dataset of human mammary epithelial (MCF10A) cells by Panchy et al^26^. The MCF10A cells were treated with increasing concentrations of TGF-β, an inducer of EMT, (0, 12.5, 25, 50, 100, 200, 400, and 800 pM, respectively) for 14 days and then sequenced, encompassing a total of 8983 cells (Fig. S1A). In the following discussion this dataset is referred to as the static MCF10A dataset. To confirm EMT progression, we calculated an EMT score for each cell across the entire range of TGF-β concentrations using a Kolmogorov-Smirnov (KS) EMT scoring method, where a negative score indicates a cell as more epithelial while a more positive score indicates a cell as more mesenchymal^27^. As the TGF-β concentration increased the KS EMT score increased indicating that cells overall gained mesenchymal characteristics and lost epithelial characteristics (Fig S1B), consistent with what was observed by Panchy et al.^26^

To visualize EMT progression in the gene expression space, we examined the single-cell data with a two-dimensional Uniform Manifold Approximation and Projection (UMAP) representation^28^ (Fig S1B). With increasing TGF-β concentrations the cells shifted from left to right, suggesting that the first UMAP mode (UMAP1) mainly reflects the TGF-β response, while the second mode (UMAP2) mostly captures TGF-β independent variations. Curiously the four highest doses (100, 200, 400, 800 pM of TGF-β) seem to settle in the same region on the 2D UMAP (Fig S1B).

Observing the cells in the 3D UMAP representation reveals that the cell population with each increasing TGF-β concentration simultaneously moves upward along UMAP3 as well as to the right along UMAP1 (Fig. S1C, D). Amongst the cells treated with the higher TGF-β concentrations, the KS EMT scores also reflect the coexistence of a heterogeneous continuum of EMT states (Fig. S1E, F), consistent with what was reported in other studies^26,29^. Overall, the 3D UMAP provides a clearer representation of EMT progression and reveals how the comprehensive perception can be obscured by the limitation and distortion that can occur in 2D representations. This observation resonates with recent debates on the common practice of analyzing single-cell data in a reduced two-dimensional space^30,31^, which we further examined in subsequent discussions.

**Figure 1.**
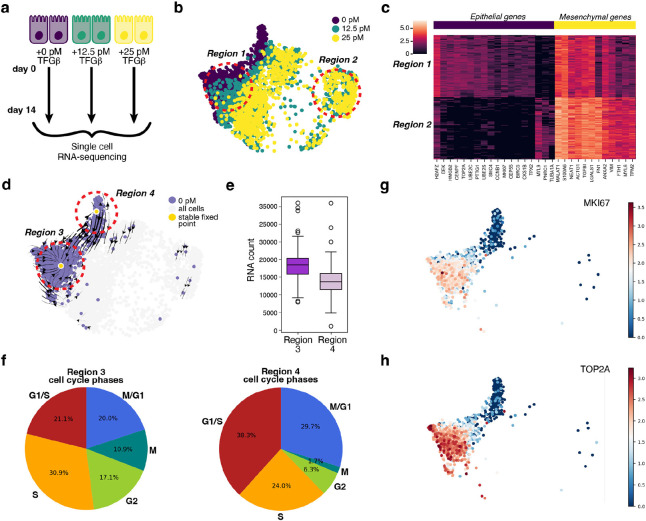
Dose-dependent TGF-β induced MCF10A cells undergo EMT continuum. (A) MCF10A cells were treated with increasing concentrations of TGF-β over the span of 14 days, pooled together and then scRNA-seq was performed. (B) The four lower doses of TGF-β (0 pM, 12.5 pM and 25 pM) projected into UMAP space. (C) Gene expression comparison between region 1 and region 2 looking at epithelial and mesenchymal marker genes. (D) Vector field of untreated MCF10A cells with stable fixed points. (E) Comparison of the mRNA count between region 1 and region 2 surrounding the stable fixed points found in the untreated vector field. (F) Comparison of the distribution of cell cycle phases between region 1 and region 2. (G, H) Mki67 and Top2a gene expression in the untreated cells.

Among the eight TGF-β concentrations, only the cells treated with 12.5 and 25 pM of TGF-β retain a subpopulation of epithelial cells indicated by the overlap with the untreated cells, which only contain epithelial cells (Fig. S1G, H). The cells treated with the other concentrations of TGF-β contain few to no epithelial cells as most cells are undergoing EMT and have already moved away from the epithelial region, occupied by the untreated cells. In subsequent analyses on understanding how cells transition from the epithelial to mesenchymal region, we focused on the two lowest TGF-β concentrations (12.5 pM and 25 pM) in addition to the untreated cells (0 pM) (Fig 1A, B). It should be noted that each TGF-β concentration for RNA velocity analysis, must be analyzed separately, as the dynamics between TGF-β concentrations cannot be combined.

To further confirm that the partial or full EMT has taken place in the low TGF-β treated cells, we compared the EMT gene expression profiles between two regions selected in the presumed epithelial and mesenchymal regions and observed a global trend (Fig 1C). Indeed, epithelial-related genes, e.g., epithelial marker genes, *Cdh1* and *EpCAM*, involved in maintaining cell-cell adhesion, and *S100A9*, had higher expression in the epithelial region than that in the mesenchymal region. Conversely, mesenchymal related genes, e.g., mesenchymal marker genes, *Vim, Fn1*, and *Cdh2*, who play a role in cell-cell adhesion and cell migration, had the reverse trend. These results are consistent with previous reports that have shown that under these TGF-β concentrations MCF10A cells undergo partial EMT^32^.

### Transcriptomic vector field of untreated MCF10A cells projected into a 2D UMAP representation reveals both quiescent and cycling cells but is biologically distortive.

To analyze the EMT process, we began with the untreated MCF10A cells. We first applied *dynamo* to obtain the corresponding RNA vector field (Fig. 1D). The reconstructed vector field identified two stable fixed points, towards which vectors converge, representing stable cell states. To characterize these two stable cell states, we selected cells around these two fixed points and performed a comparative analysis between the two regions. Interestingly, cells in region 3 had significantly higher raw mRNA counts than cells in region 4 (independent sample T-test *p* value 9e^−19^) (Fig 1E). Further, cell cycle phase estimation analysis revealed that region 4 had 17.1 percent more cells in the G1/S phase and 9.7 percent more cells in the M/G1 phase than in region 3 (Fig 1F). In addition, the gene expression of cell cycle genes *Mki67*, a marker for proliferation, and *Top2a*, a gene encoding a DNA topoisomerase, have higher expression in region 3 than in region 4 (Fig 1G, H). Collectively, these results suggest that region 3 corresponds to cells actively proliferating while region 4 corresponds to cells enriched in the quiescent (i.e., G0) state.

Although the 2D UMAP reduced representation of the untreated cells was able to correctly identify two subpopulations of cells, quiescent and proliferating, the representation distorts the cell cycle dynamics. Specifically, the region 3 stable fixed point, identified on the 2D space is both biologically and mathematically unreasonable, as it represents actively proliferating cells, which is not a stable state. Instead, one would expect to observe circulating vectors to represent proliferating cells. To be more specific, with proliferative cells one would expect that the single-cell data form a geometric manifold containing at least one hole reflecting cell cycle progression in the cell state space.

**Figure 2.**
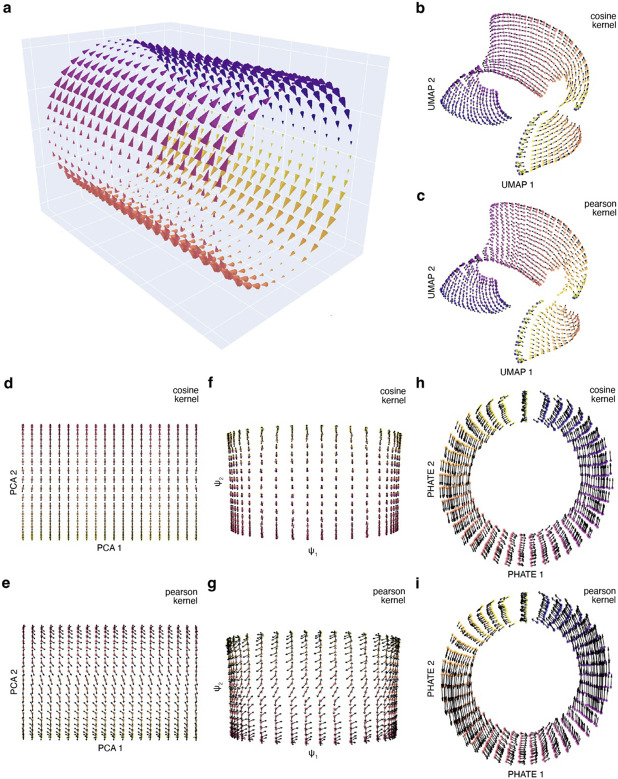
Dimension reduction from 3D to 2D distorts the dynamics. (A) 3D cylindrical vector field. (B, C) The 3D vector field projected to 2D UMAP space using cosine or pearson kernel. (D, E) The 3D vector field projected to 2D PCA space using cosine or pearson kernel. (F,G) The 3D vector field projected to 2D diffusion map space using cosine or pearson kernel. (H,I) The 3D vector field projected to 2D PHATE space using cosine or pearson kernel.

A simplified representation of this would be a cylindrical velocity vector field, where the cell cycle is depicted by the circle of the cylinder, and additional processes are depicted as an extension off the circle, forming a cylindrical structure (Fig. 2A). Such a geometric manifold is topologically impossible to be faithfully represented in a two-dimensional space without slicing or folding the manifold onto a flat surface. This was exemplified when different dimensionality reduction methods, Uniform Manifold Approximation and Projection^28^ (UMAP), Principal Component Analysis (PCA), diffusion Map and Potential of Heat-diffusion for Affinity-based Trajectory Embedding^33^ (PHATE) was applied to a 3D cylindrical vector field. The 2D UMAP representation heavily distorts the manifold (Fig 2B, C). When the vector field was projected into 2D using either cosine or Pearson kernel, common methods to project high dimensional vector fields to a lower dimension, it resulted in regions in the UMAP space where vectors converge revealing how fixed points can result in 2D space. In the case of PCA or diffusion map the resulting 2D representations are only able to capture one side of the cylinder (Fig 2D, E, F, G).

In this simplistic case, we have the benefit of knowing the shape of the manifold, a cylinder, and therefore can deduce why there are conflicting vector directions. However, with real scRNA-seq data the high-dimensional manifold is unknown and thus it is difficult to understand what is occurring within the data with just the reduced representations. This discrepancy is primarily attributed to the challenges of dimensionality reduction—transitioning from the high-dimensional gene expression space to a two-dimensional representation. In the process, certain sections of the high-dimensional manifold end up being folded and compressed, obscuring biological processes and distorting the velocity vector field.

PHATE was able to capture the circle in the manifold and partially capture the depth of the cylinder (Fig 2H, I). In addition, the vectors reflect the cylindrical vector field despite the reduced representation. The cylindrical vector field, however, is a simplified example of what the scRNA-seq data manifold could look like and thus more complex manifolds would pose a challenge. In conclusion, caution should be exercised when interpreting scRNA-seq data using reduced representations.

### Two-dimensional transcriptomic vector field reveals two parallel EMT paths in TGF-β induced EMT of MCF10A cells.

**Figure 3.**
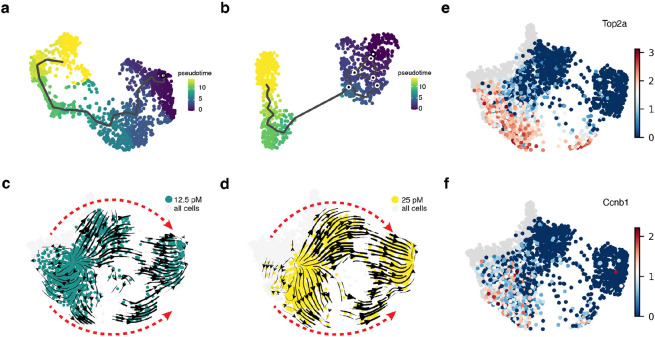
Vector fields of TGF-β induced MCF10A cells reveal two EMT paths. (A, B) Pseudotime analysis of the 12.5 and 25 pM TGF-β treated cells on UMAP space. (C, D) Vector fields of 12.5 and 25 pM TGF-β treated cells on UMAP space. The red arrows indicate the two EMT paths. (E, F) Top2a and Ccnb1 gene expression for both the 12.5 and 25 pM TGF-β treated cells on UMAP space.

A fundamental question in the field of EMT is whether cells undergoing EMT follow a single defined path or multiple paths^15^. To investigate this question, we first applied the pseudo-time analysis method, Monocle 3^34^, to the MCF10A cells treated with 12.5 and 25 pM of TGF-β. This analysis identified a single EMT path (Fig 3A, B), consistent with previous reports using pseudo-time analyses^22^ and Markovian transition model analyses^23^ on snapshot single-cell data but differs from reports from live-cell imaging studies^24,25^.

**Figure 4.**
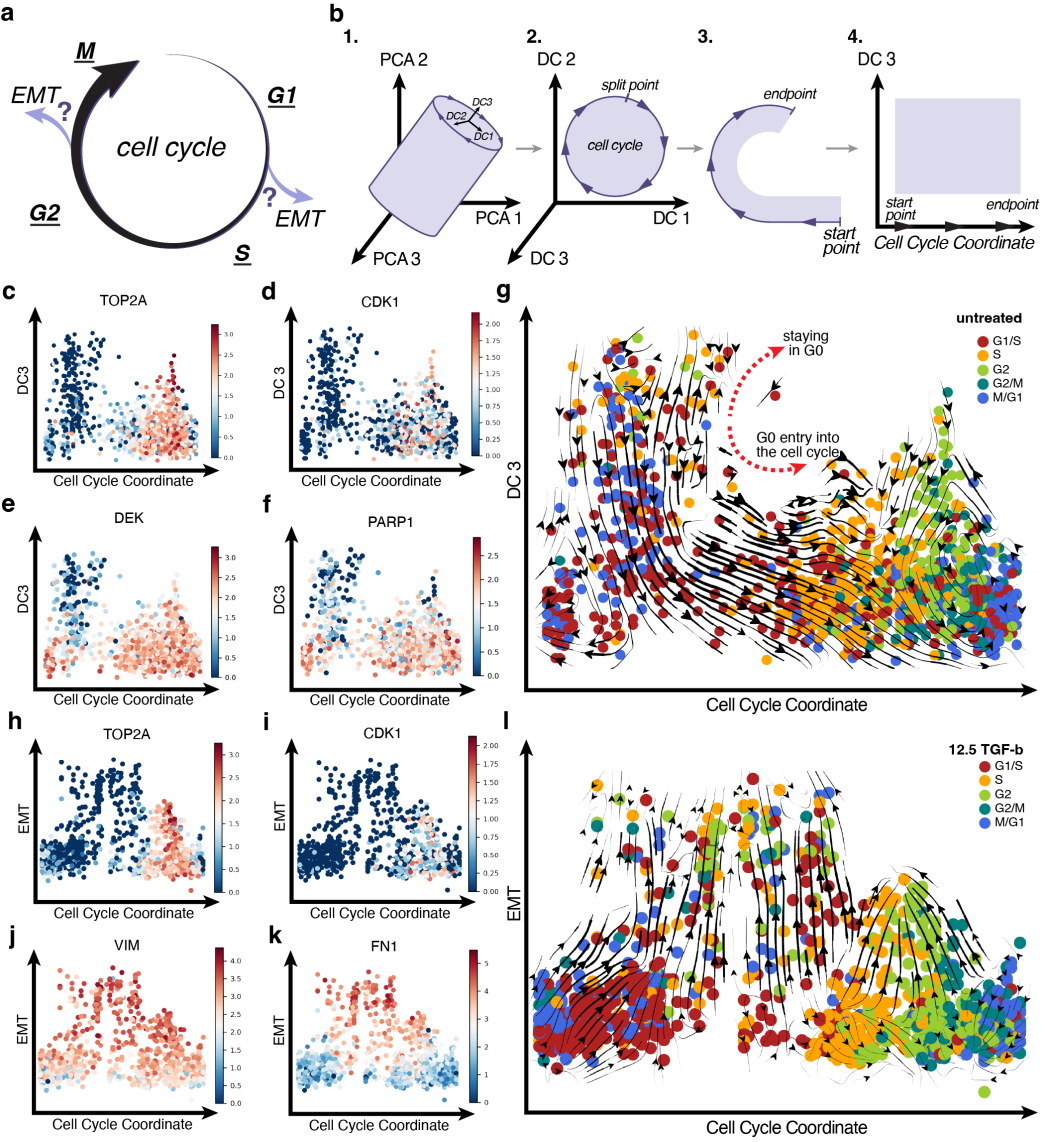
The cell cycle-EMT representation reveals two paths. (A) Cell cycle coupling with EMT hypothesis. (B) The computational pipeline to obtain the Cell Cycle Coordinate. (C, D) Top2a and Cdk1 gene expression to confirm cell cycle progression along the x-axis. (E, F) Dek and Parp1 gene expression confirming quiescence entry into the cell cycle along the y-axis. (G) The cell cycle-quiescent representation obtained from the untreated MCF10A cells reveal quiescent cell entry into the cell cycle. (H, I) Top2a and Cdk1 cell cycle gene expression to confirm cell cycle progression along the x-axis. (G) Vim and Fn1 EMT gene expression to confirm the EMT progression along the y-axis. (L) The cell cycle-EMT representation obtained from the 12.5 pM TGF-β treated MCF10A cells reveal two paths.

To investigate this discrepancy further, we performed vector field analysis using *dynamo* on the MCF10A cells treated with 12.5 and 25 pM of TGF-β . Notably, each vector field demonstrated a consistent pattern of EMT progression, with vectors shifting from left to right through two visually distinct transitions paths (Fig 3C, D). We noticed that several G2 cell cycle phase related genes, *Top2a* and *Ccnb1*, exhibited higher expression in the lower group of cells across the two concentrations of TGF-β treated cells (Fig 3E, F). This observation led us to hypothesize that the upper and lower EMT paths observed from the vector fields were due to the cell cycle coupling with EMT at the G1/S and G2/M checkpoints (Fig 4A).

However, it is unclear how the cell cycle couples with EMT in the current two-dimensional vector fields obtained from the TGF-β treated cells. To test our cell cycle-EMT coupling hypothesis we required a representation that could simultaneously reflect cell cycle progression as well as EMT status of individual cells.

### Computational pipeline identifies the Cell Cycle Coordinate.

To determine whether the cell cycle and EMT couple together we required a representation that takes cell-cycle information into account, which is not explicitly accounted for in widely used dimensionality reduction methods. Therefore, we developed a four-step pipeline that can generate a biologically meaningful axis that captures cell cycle progression (Fig 4B).

The pipeline utilizes the raw spliced mRNA counts from scRNA-seq data as input to Revelio^35^, an R package (Fig 4B subpanel 1). Revelio assumes that the proliferative cells approximately reside on a hollow hypercylinder manifold. In step one, Revelio represents the data in high-dimensional principal component (PC) space and then rotates the coordinate frame to define new axes referred to as dynamical components (DC). The first two DCs, DC1 and DC2 respectively, of the high-dimensional DC space encapsulate most of the cell cycle-dependent variation. In step two, we used an iterative finite temperature string method^24^ to obtain a one-dimensional cyclic Cell Cycle (CC) coordinate on the DC1-DC2 plane (Fig 4B subpanel 2). Next, we identified the cell cycle division point, the split point, where we then unraveled the Cell Cycle coordinate to form a linear axis with a periodic boundary condition reflecting cell cycle progression (Fig 4B subpanel 3 and 4). The remaining DCs orthogonal to the DC1-DC2 plane contain variations in the data that are mostly cell cycle-independent. Thus, in our subsequent analysis, we employed the CC Coordinate and either the third dynamical component, DC3, or a combination of the non-DC1/DC2 (i.e. DC3 and onward) dynamical components to form a low-dimensional representation describing cell cycle progression and other cellular processes within the data (Fig 4B subpanel 4). Details are provided in the method section.

### The cell cycle representation reveals cycling and quiescent dynamics.

We first applied this computational pipeline to the untreated MCF10A cells. Indeed, single-cell data on the DC1-DC2 plane captured the cyclical nature of the cell cycle, which is further reflected in the vector field (Fig S2 A, B). The obtained CC coordinate accurately captures cell cycle progression, with the estimated cell cycle phase calculated for each cell reflecting the sequential cell cycle progression (Fig S2C). As an additional check, the gene expression profiles of cell cycle related genes, *Top2a* and *Cdk1*, showed expected elevated expression at the G2/M phase of the CC coordinate (Fig 4C, D). Moreover, the proliferating region identified in the UMAP space, region 3, in the new CC representation was spread across the cell cycle (Fig S2D, E). In addition, the previously identified quiescent region, region 4, in the new CC representation was concentrated in the upper region of the DC3 axis connecting with the G1/S phase of the cell cycle, marking the transition from quiescence into the cell cycle (Fig S2D, E). Furthermore, the gene expression of *Dek* and *Parp1*, genes identified as universally down-regulated during quiescence^36^, had reduced expression in the identified quiescent region and higher expression in the actively proliferating region (Fig 4E, F).

The vector field obtained from the untreated MCF10A cells using the CC-DC3 representation further provides a dynamic view of cell cycle progression revealing a bifurcation between proliferation and quiescence (Fig 4G). Above the bifurcation, the vector field flows upward indicating cells progressing deeper into quiescence, while below the bifurcation, the vector field flows downward signaling entry into the cell cycle. Such bifurcation reflects the quiescence-proliferation transition controlled by a Rb-E2F bistable switch: after mitosis the daughter cells either enter quiescence spontaneously or proceed to the G1 phase of the cell cycle^37^. In conclusion, the CC-DC3 representation correctly captures quiescence-proliferation dynamics.

### The cell cycle-EMT representation reveals how the cell cycle and EMT couple together.

Next, we applied our computational pipeline to the 12.5 pM TGF-β treated MCF10A cells. Consistent with what was observed with the untreated cell data, the CC coordinate accurately depicts cell cycle progression, corroborated by the high expression of *Top2a* and *Cdk1* in the G2 and G2/M phase (Fig 4H, I, Fig S3F). Gene expression analysis also confirmed that the new y-axis indeed effectively captures EMT progression, with increasing gene expression levels of *Vim* and *Fn1*, mesenchymal related genes (Fig 4J, K). That is, as cells move upward along the EMT axis, they gradually lose their epithelial features and gain mesenchymal features.

Like the 2D UMAP representation, from the vector field on the CC-EMT representation one can visually identify two types of EMT paths (Fig 4L, Fig S2F). The first path begins with epithelial cells in the G1/S phase, where they then exit the cell cycle and proceed through EMT. The second path begins with epithelial cells in the G2 or G2/M phase, where they then exit the cell cycle, and proceed through EMT.

When the computational pipeline was applied to the 25 pM TGF-β treated MCF10A cells we observed the same G1/S and G2/M paths found in the 12.5 pM TGF-β treated cells in addition to another path potentially corresponding to cells arrested in the M phase (Fig S2G, H, I, J, K). The emergence of this additional path suggests that MCF10A cells respond to TGF-β in a dose dependent manner, with higher concentrations inducing greater cellular arrest, due to increased DNA damage, driving cells to undergo M cell cycle arrest leading to three distinct paths cells can take to undergo EMT.

Overall, our computational pipeline obtains a representation that captures cell cycle progression along a single axis which enables the extraction of valuable biological insight regarding the coupling between the cell cycle and EMT.

### Trajectory simulations reveal two EMT paths in multi-dimensional state space.

**Figure 5.**
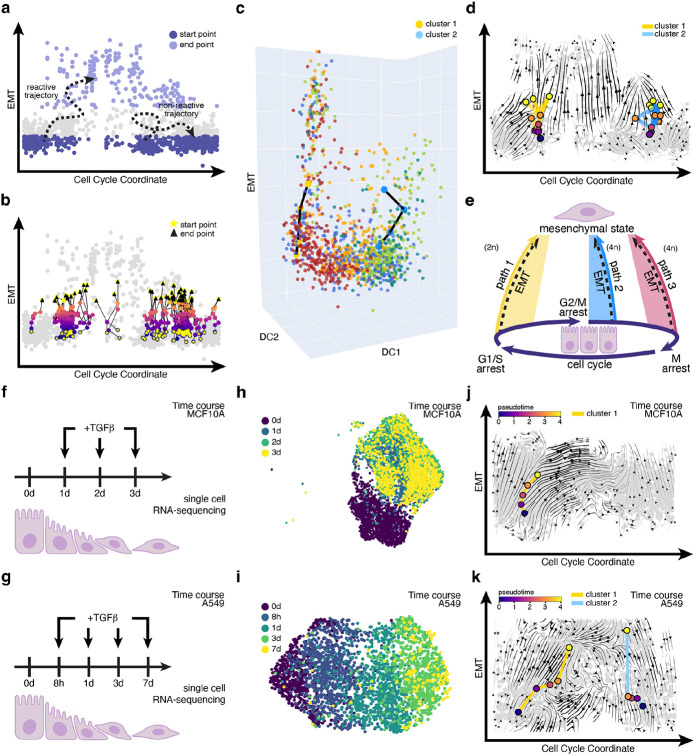
Paths occur in high dimensions and in other datasets. (A) The trajectory simulation set-up with a specified start and end region with the 12.5 pM TGF-β treated MCF10A cells. (B) One set of trajectories mapped onto the EMT/cell cycle coordinate representation. (C) The average trajectories for the two clusters mapped on to 3D space. (D) Representative trajectories mapped to the EMT/cell cycle coordinate representation. (E) Overview of the different paths and how the cell cycle and EMT process couple together. (F) Overview of the time course MCF10A scRNA-seq dataset and (G) time course A549 scRNA-seq dataset. (H) The time course MCF10A dataset and (I) the time course A549 dataset projected to UMAP space. The cell cycle-EMT representation for the (J) time course MCF10A scRNA-seq dataset and (K) the time course A549 scRNA-seq dataset with the corresponding representative trajectories

To ensure that the multiple identified EMT paths were not an artifact of dimensionality reduction and to quantify the paths, we performed trajectory simulations and transition path analyses in 30-dimensional PCA space, where the 12.5 pM vector field was originally constructed. To obtain trajectories in high-dimensional space, we first identified an epithelial and mesenchymal region from the CC-EMT representation, designating them as potential start and end points, respectively (Fig 5A). Using the transition matrix derived from the scRNA-seq data we launched trajectories from the epithelial start region and recorded trajectories that hit the mesenchymal end region, referred to as reactive trajectories. For the 12.5 pM TGF-β treated cells, we conducted ten rounds of trajectory simulations, generating 100 trajectories per simulation (Fig 5B) and found that they clustered into two distinct clusters. With these two clusters of trajectories, we used a modified finite temperature string method to obtain the mean path, similar to that of reaction coordinates in chemical reaction dynamics, per cluster, where we observed the two clusters originating from either the G1 or G2 phase (Fig 5C). Projecting this ensemble of trajectories to the CC–EMT representation, we indeed see that the two clusters start from either the G1 or G2 phase (Fig 5D). This suggests that G1/S and G2/M arrest is associated with EMT.

For the MCF10A cells treated with 25 pM TGF-β, we also performed ten rounds of trajectory simulations where we identified three clusters of trajectories (Fig S3A). We generated the mean paths for these clusters and observed that the trajectories align with those originating from either the G1 or G2 phase, similar to 12.5 pM TGF-β treated cells, or the M phase, as predicted in Fig 4I (Fig S3B).

Overall, the trajectory simulation analyses confirmed that two types of EMT paths exist in high dimensional space for cells treated with lower concentrations of TGF-β (12.5 pM). In contrast, at higher concentrations of TGF-β (25 pM) cells undergo greater cell cycle arrest, possibly due to increased DNA damage, leading to a third path corresponding to M cell cycle arrest.

To summarize the two paths found in cells treated with the lower concentration of TGF- β, cells following G1/S path start in the epithelial state in G1, then undergo G1/S arrest and undergo EMT (Fig 5E). On the other hand, cells following G2/M are cells that have already passed the G1/S checkpoint and have continued onward to the G2/M checkpoint where the cells undergo cell cycle arrest and then EMT. However, at higher concentrations of TGF-β, the two previously mentioned paths (G1 and G2) persist but a third path emerges when cells accrue excessive DNA damage preventing them from passing the M cell cycle checkpoint, resulting in M cell cycle arrest.

### Time course scRNA-seq MCF10A and A549 cells treated with TGF-β reveal paths.

To investigate whether the coupling observed between the cell cycle and EMT can be found in other contexts, we expanded our investigation to time course scRNA-seq datasets of other cell lines undergoing TGF-β induced EMT. We intentionally selected datasets with diverse experimental setups to determine whether the presence of the two observed paths in the static MCF10A dataset could be identified across different contexts. The datasets include a time course dataset of MCF10A treated with 200 pM of TGF-β^38^ and another one of adenocarcinomic human alveolar basal epithelial (A549) cells treated with 400 pM TGF-β^39^. The time course MCF10A scRNA-seq dataset consisted of cells undergoing TGF-β induced EMT over the span of 3 days (0d, 1d, 2d, 3 d) totaling to 5481 cells (Fig 5F). The time course A549 scRNA-seq dataset consisted of cells undergoing TGF-β induced EMT, over the span of seven days (0d, 8h, 1d, 3d, and 7d) totaling to 3132 cells (Fig 5G). The UMAP representations for both time course datasets broadly depict EMT progression (Fig 5H, I, Fig S4A, B), but does not provide a clear picture of how the cell cycle couples with EMT.

After applying our computational pipeline to capture the cell cycle, the resulting CC-EMT representation for each dataset showed a clear alignment along the EMT axis with the datasets increasing timepoints (Fig S4C, D). The gene expression profile of the cell cycle gene marker, *Top2a*, indicates that the CC coordinate captures cell cycle progression (Fig S4E, F). In addition, the expression profiles of EMT gene markers, *Vim* and *Fn1*, confirm that the y-axis represents EMT (Fig S4G, H, I, J). This confirms that the CC-EMT representation indeed represents both the cell cycle and EMT.

With the CC-EMT representation vector field analysis and trajectory simulations were performed. For the time course MCF10A dataset only a single path corresponding to the G1/S path was identified (Fig 5J, Fig S4K). For the A549 dataset we identified two paths corresponding to the G1/S and G2/M paths, with the former dominating (~60 %) (Fig 5K, Fig S4L). To ensure that there was not a bias for the number of specified clusters, for the time course MCF10A dataset we tested for one and two clusters but was only able to identify the same G1/S path cluster as the dominant one (Fig S5A, B). For the time course A549 dataset we tested for two, three, and four clusters but were only able to identify two paths (Fig S5C, D, E, F). These results together with the results from the static MCF10A analysis suggest that at lower concentrations of TGF-β in MCF10A cells the G1/S and G2/M path exists, while at much higher concentrations of TGF-β the G1/S path completely dominates. Meanwhile in a different context, the two paths seem to coexist in A549 cells at high TGF-β concentrations.

With the CC-EMT representation vector field analysis and trajectory simulations were performed. For the time course MCF10A dataset only a single path corresponding to the G1/S path was identified (Fig 55JK, Fig S45K). For the A549 dataset we identified both two paths corresponding to the G1/S and G2/M paths, with the former dominating (~60 %) (Fig 5KL, Fig S45L). To ensure that there was not a bias for the number of specified clusters, for the time course MCF10A dataset we tested for one and two clusters but was only able to identify the same G1/S path cluster as the dominant one (Fig S56A, B). For the time course A549 dataset we tested for two, three, and four clusters but were only able to identify two paths (Fig S56C, D, E, F).

These time course results suggest, along with the static MCF10A analysis, suggests that at lower concentrations of TGF-β, MCF10A cells follow either the G1/S or G2/M path. As TGF-β concentration increases, an additional path M cell cycle arrest path emerges. However, at much higher concentrations of TGF-β cells the cell cycle almost completely inhibited, resulting in the dominance of a single G1/S path. In contrast, in a different cellular context, in A549 cells the two paths seem to coexist even in at high TGF-β concentrations. Overall, this speaks to the heterogeneity nature of cellular responses to TGF- β.

## Discussion

When a proliferating cell undergoes cell phenotypic transition, it typically needs to temporarily or permanently exit the cell cycle^4^. Considering the existence of cell cycle checkpoints, it is natural to expect that cell exit takes place at only a few restricted regions of the cell cycle. Analyzing single cell transcriptomic data of cells undergoing EMT, however, reveals that the exit points are broadly distributed along the cell cycle coordinate. One mechanistic explanation is that initiation of EMT is largely decoupled to cell cycle progression until the suppression of EMT on the latter reaches a threshold value for a cell cycle checkpoint regulatory program, leading to a distribution of the exit points and contributing to the observed EMT heterogeneity. Furthermore, the suppression of EMT on the cell cycle is different for different cell cycle checkpoints and is stimulus-dose-dependent and cell-type-dependent. This observation emphasizes that biomedical intervention of cell phenotypic transitions requires quantitative understanding of its crosstalk to cell cycle progression.

To perform the single-cell data analyses, a standard procedure is applying dimensionality reduction to high dimensional data to obtain a two-dimensional representation, as it helps to visualize such complex data. For example, many trajectory inference approaches are typically performed on low-dimensional representations of single-cell data, and thus require that the representation faithfully reflects the cellular processes being studied. Single-cell omics approaches characterize cell state heterogeneity by quantifying the transcriptomic, proteomic, or epigenomic profiles of individual cells. Thus, a grand challenge is how to identify cellular processes relevant to a specific question of interest. A representation obtained with a *brute force* dimensionality reduction procedure, which emphasizes the largest contributions to heterogeneity, may conceal or distort the impact from relevant processes, such as the cell cycle, as shown in this study. Furthermore, most dimensionality reduction methods do not include dynamic information, further worsening the situation.

In this study, we demonstrated the importance of examining cellular dynamics in a high dimensional representation, aided by dynamical information such as a transcriptomic vector field, to reduce mechanistic ambiguity. Additionally, when visualizing data in low-dimensional representations, incorporating biologically informed information to the representation aids in understanding how processes couple. With the full dynamical model using RNA velocity information, we performed single-cell trajectory simulations in a high-dimensional state space, which confirmed the existence of multiple classes of trajectories. Note that the single-cell trajectories here are different from “trajectory” inference conventionally used in the single-cell field. The latter typically refers to mean trajectories followed by an ensemble of cells that corresponds to the mean paths discussed in this work. In our case, single-cell trajectories refer to trajectories on a single-cell level.

This study demonstrates how varying representations can reveal different mechanistic insights into cellular processes from single-cell data, not to provide an optimal representation. We also restrained this study from systematic investigations on how EMT couples with the cell cycle and other processes. Future studies can expand along several directions. We followed the Revelio model by assuming that one can approximate the single-cell data manifold to be cylindrical-shaped to describe cell cycle-dependent and -independent variations. However, the data manifold is expected to be curved and this assumption is only valid locally. An improved representation with explicit cell cycle description would be beneficial. In addition, we restricted our transition path analyses to a specific final mesenchymal state. The MCF10A data at high concentrations of TGF-β suggests the coexistence of different mesenchymal states, thus more than two types of EMT transitions paths may exist. Moreover, one can extend the trajectory analyses to investigate the coupling between various cellular processes such as EMT, gain or loss of stemness, and decisions among various cellular fates such as apoptosis and senescence. Systematic identification of these different transition processes may require trajectory analyses without pre-selected start and end states. In short, with further improved cell state resolution and data sampling, combined experimental and biology-informed in-silico trajectory analyses can help to provide systematic characterization and mechanistic insight into how different cellular processes couple together to respond to one or multiple environmental and intracellular stimuli.

## Supplementary Material

Supplement 1

## Data Availability

All the raw sequencing data for the static MCF10A dataset is available in the NCBI Gene Expression Omnibus under the accession GSE213753. All the raw sequencing data for the MCF10A time course dataset is available at the National Center for Biotechnology Information Sequence Read Archive (BioProject ID: PRJNA698642). All the raw sequencing data for the A549 time course dataset is available in the NCBI Gene Expression Omnibus under the accession GSE147405.

## Code availability

All code can be found on GitHub https://github.com/xing-lab-pitt/two-paths.
