# Supplementary material for: Epithelial-mesenchymal transition couples with cell cycle arrest at various stages
Source: bioRxiv. 2025 Feb 28:2025.02.24.639880. Preprint. [Version 1] doi: 10.1101/2025.02.24.639880 (PMC11888286; doi:10.1101/2025.02.24.639880)
Supplement: Supplement 1 [file NIHPP2025.02.24.639880v1-supplement-1.pdf]

bioRxiv preprint doi: https://doi.org/10.1101/2025.02.24.639880; this version posted February 28, 2025. The copyright holder for this preprint (which was not certified by peer review) is the author/funder, who has granted bioRxiv a license to display the preprint in perpetuity. It is made available under aCC-BY 4.0 International license.

Supplemental Figures:

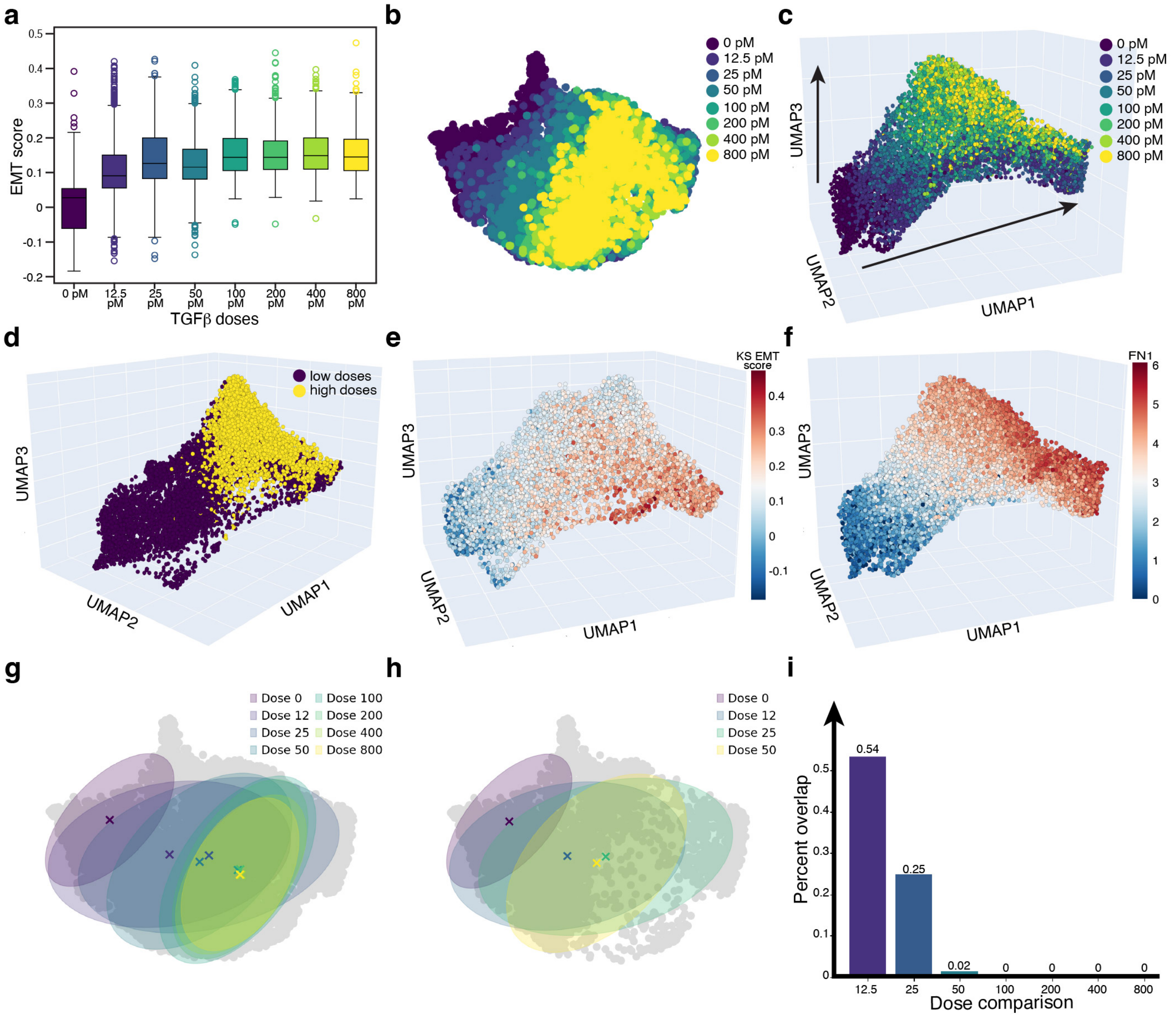

Supplemental Figure 1. Analyses of scRNA-seq datasets of TGF-β treated MCF10A cells. *(A) Epithelial (E) and mesenchymal (M) scores for cells treated with increasing concentrations of TGF-β. (B) scRNA-seq data of cells treated all TGF-β concentrations (0 pM, 12.5 pM, 25 pM, 50 pM, 100 pM, 200 pM, 400 pM, 800 pM) projected into two-dimensional UMAP space. Colors represent TGF-β concentrations. (C) scRNA-seq data shown in the leading three-dimensional UMAP space. Colors represent TGF-β concentrations. (D-F) Same as panel C, colored by KS EMT score (lower KS score = more epithelial and higher KS score = more mesenchymal) and Fn1 gene expression, respectively. (G)(H)(I) The percent area overlap each TGF-β concentration has with the untreated MCF10A cells.*

bioRxiv preprint doi: https://doi.org/10.1101/2025.02.24.639880; this version posted February 28, 2025. The copyright holder for this preprint (which was not certified by peer review) is the author/funder, who has granted bioRxiv a license to display the preprint in perpetuity. It is made available under aCC-BY 4.0 International license.

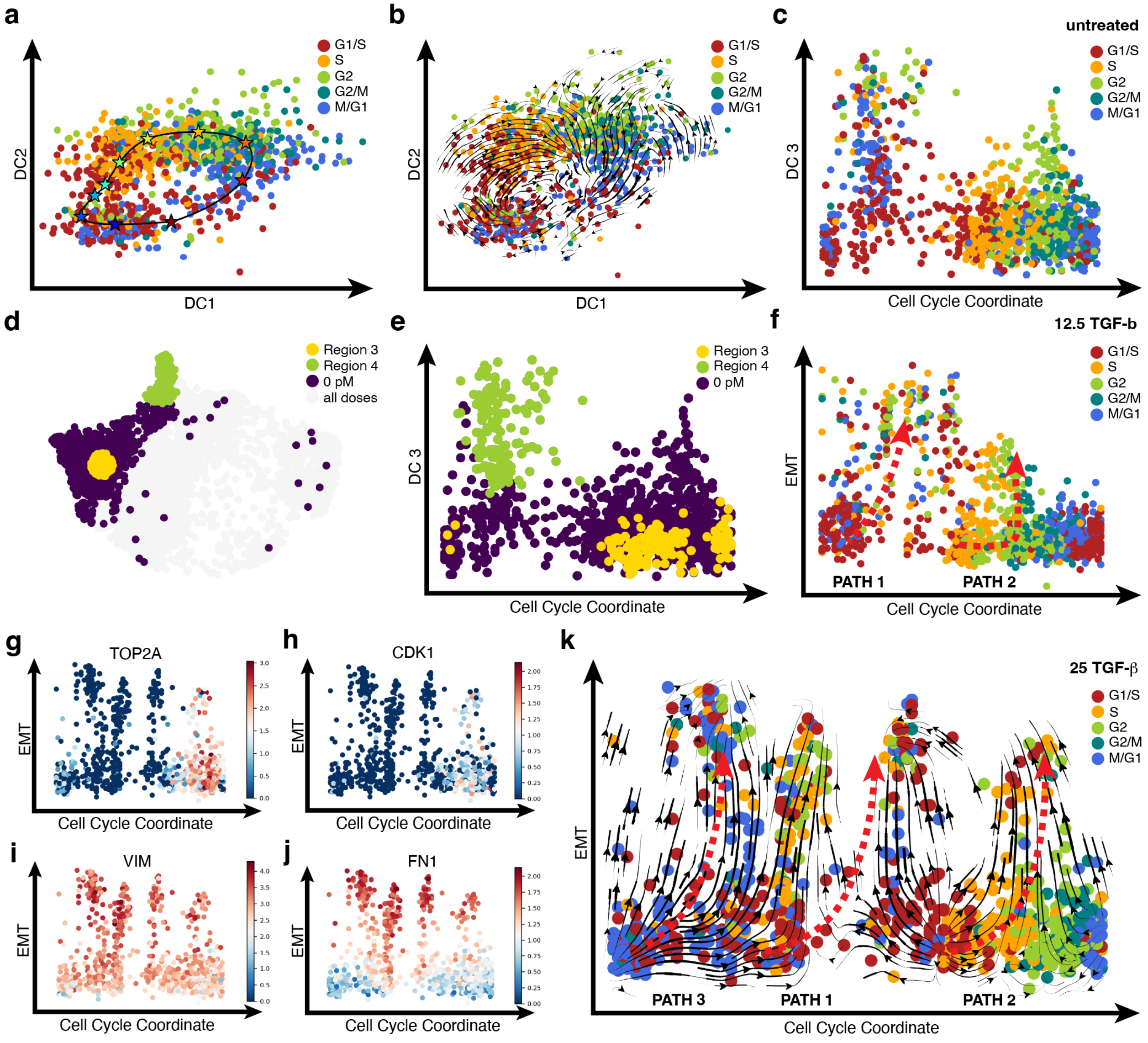

Supplemental Figure 2. *(A) Untreated MCF10A cells in DC1-DC2 space colored by the estimated cell cycle phase. The initial cell cycle coordinate is represented by the line with stars. (B) The vector field obtained with the untreated MCF10A cells in DC1-DC2 space colored by estimated cell cycle phase. (D) Untreated MCF10A cells regions in 2D UMAP space. The colors indicate the cells chosen for differentiation gene expression analysis. (E) Untreated MCF10A cells in Cell Cycle Coordinate-EMT space colored by the corresponding regions. (F) MCF10A cells treated with 25 pM of TGF-β in Cell Cycle Coordinate-EMT space colored by cell cycle phase along with the corresponding vector field. The two paths are marked by the red dashed arrows (G, H) The MCF10A cells treated with 25 pM of TGF-β in Cell Cycle Coordinate-EMT space colored by Top2a and Cdk1 expression. (I, J) The MCF10A cells treated with 25 pM of TGF-β in Cell Cycle Coordinate-EMT space colored by Vim and Fn1 expression. (K) Vector field of dose 25 TGF-β MCF10A treated cells. The dashed arrows indicate the different paths.*

bioRxiv preprint doi: https://doi.org/10.1101/2025.02.24.639880; this version posted February 28, 2025. The copyright holder for this preprint (which was not certified by peer review) is the author/funder, who has granted bioRxiv a license to display the preprint in perpetuity. It is made available under aCC-BY 4.0 International license.

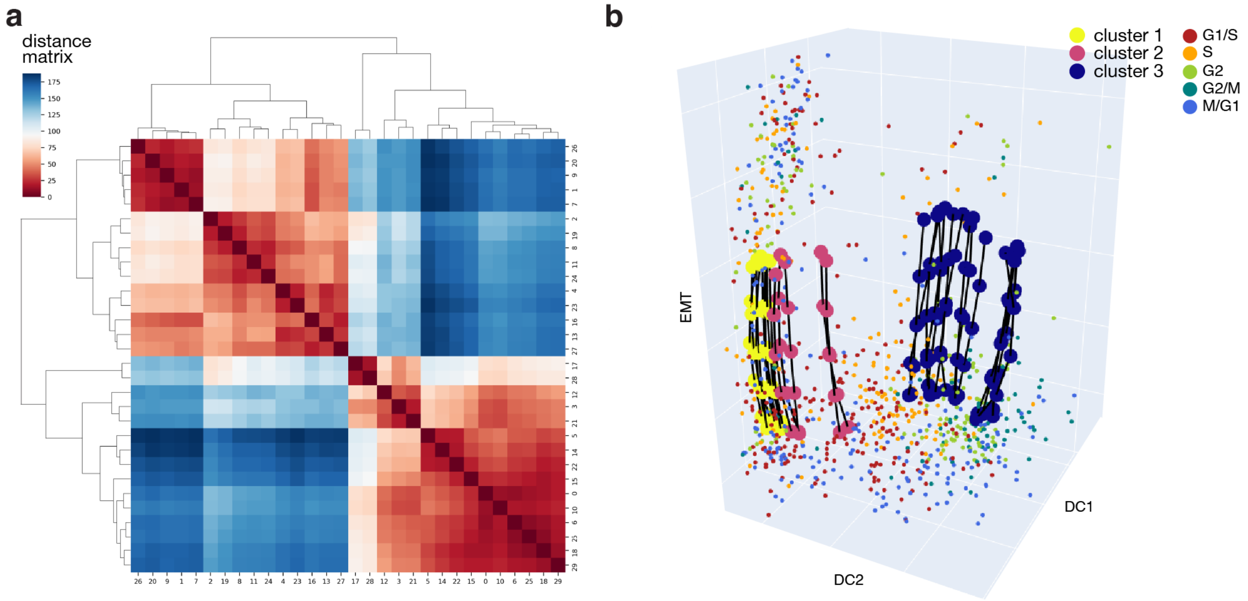

Supplemental Figure 3. *(A) Distance matrix of the 30 representative trajectories from trajectory simulations. (B) Thirty representative trajectories mapped into 3D space.*

bioRxiv preprint doi: https://doi.org/10.1101/2025.02.24.639880; this version posted February 28, 2025. The copyright holder for this preprint (which was not certified by peer review) is the author/funder, who has granted bioRxiv a license to display the preprint in perpetuity. It is made available under aCC-BY 4.0 International license.

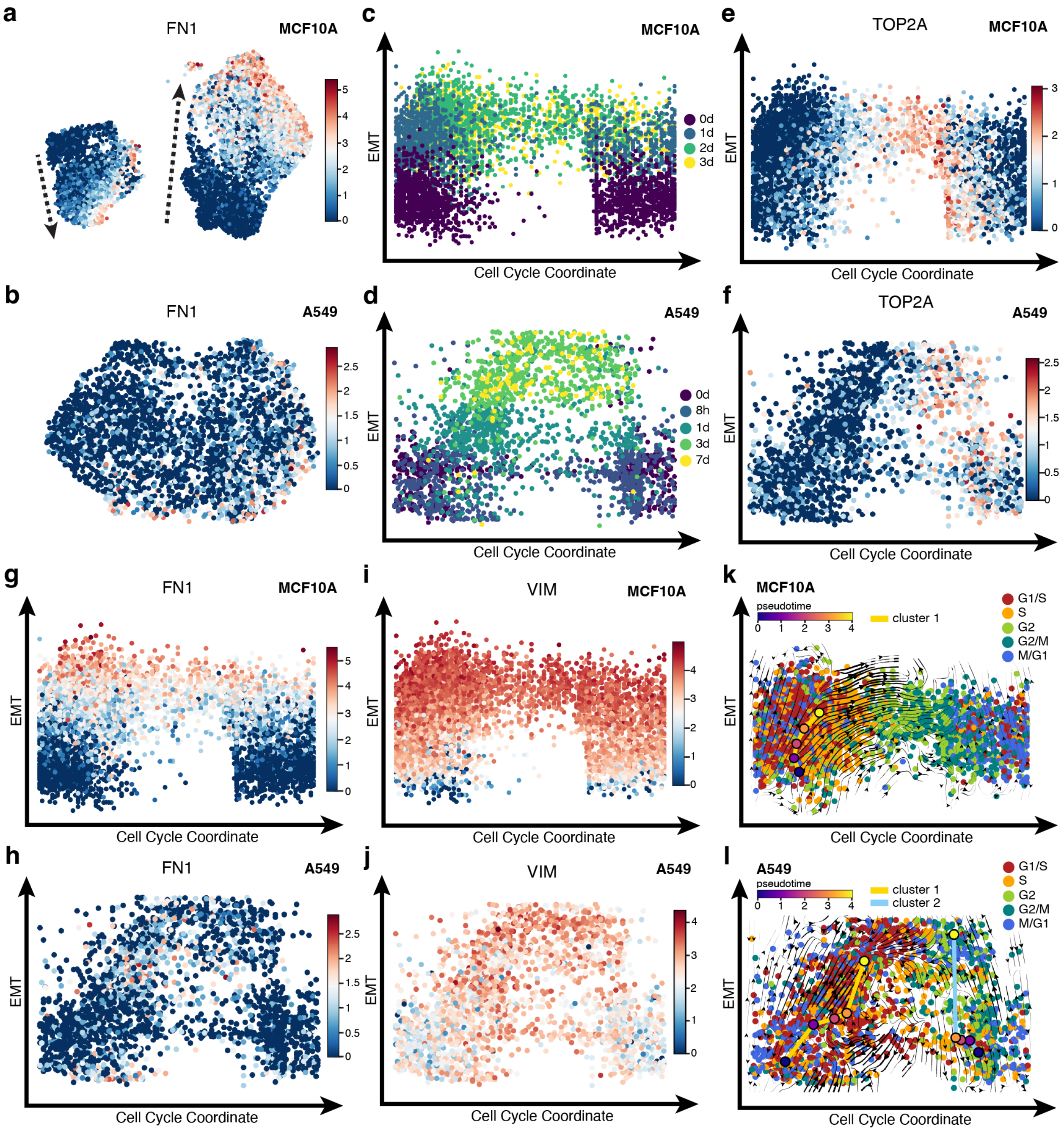

Supplemental Figure 4. *(A) Time course MCF10A dataset in UMAP space colored by FN1 gene expression. The arrows indicate two groups of EMT progression. (B) Time course A549 dataset in UMAP space colored by FN1 gene expression. (C, D) Time course MCF10A and A549 datasets in Cell Cycle Coordinate-EMT space colored by timepoint. Time course MCF10A and A549 datasets in Cell Cycle Coordinate-EMT space colored by (E, F) Top2a, (G, H) Fn1, (I, J) Vim gene expression. (K, L) Time course MCF10A and A549 datasets in Cell Cycle Coordinate-EMT space colored by cell cycle phase along with the corresponding vector fields and mean trajectories representing the paths identified during the trajectory simulations.*

bioRxiv preprint doi: https://doi.org/10.1101/2025.02.24.639880; this version posted February 28, 2025. The copyright holder for this preprint (which was not certified by peer review) is the author/funder, who has granted bioRxiv a license to display the preprint in perpetuity. It is made available under aCC-BY 4.0 International license.

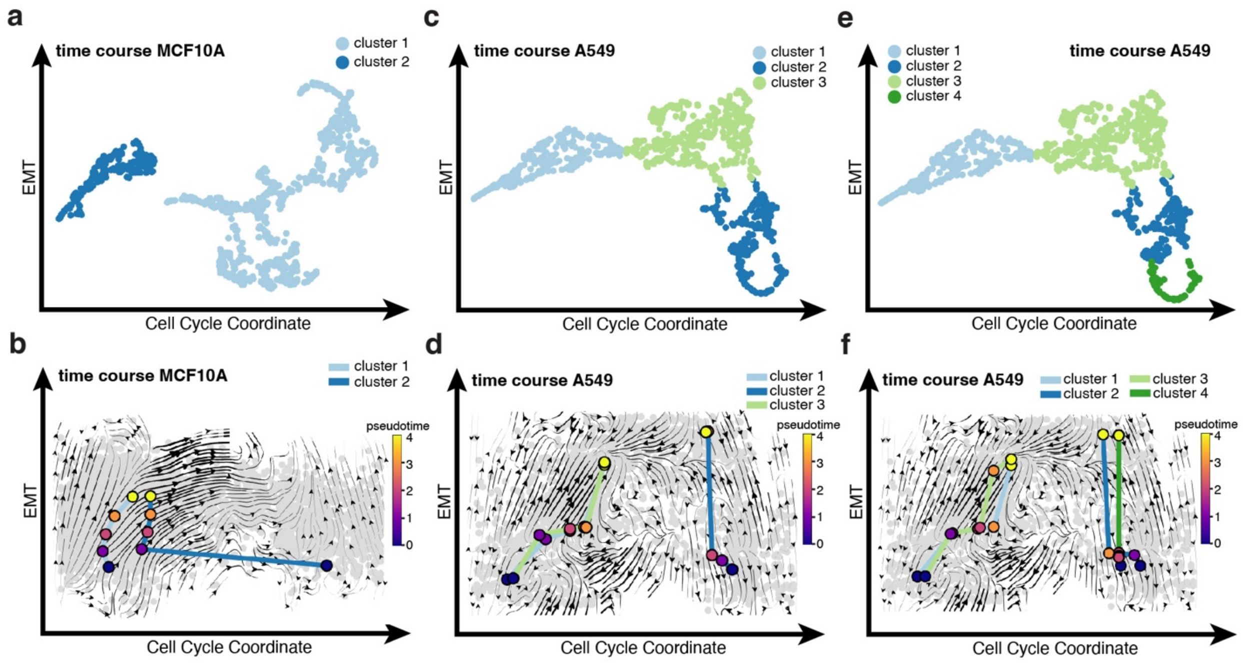

Supplemental Figure 5. *(A) The time course MCF10A trajectories clustered into two groups. (B) The two clusters mapped on the Cell Cycle Coordinate-EMT representation for the time course MCF10A cells. (C, D) The time course A549 trajectories clustered into three groups along with the clusters mapped onto the corresponding CC-EMT representation. (E, F) The time course A549 trajectories clustered into four groups along with the clusters mapped onto the corresponding CC-EMT representation.*
